# Feasibility of hair sampling to assess levels of organophosphate metabolites in rural areas of Sri Lanka

**DOI:** 10.1016/j.envres.2016.02.011

**Published:** 2016-05

**Authors:** D.W. Knipe, C. Jayasumana, S. Siribaddana, C. Priyadarshana, M. Pearson, D. Gunnell, C. Metcalfe, M.N. Tzatzarakis, A.M. Tsatsakis

**Affiliations:** a School of Social and Community Medicine, University of Bristol, Canynge Hall, 39 Whatley Road, Bristol BS8 2PS, UK; b South Asian Clinical Toxicology Research Collaboration (SACTRC), Faculty of Medicine, University of Peradeniya, Peradeniya, Sri Lanka; c Department of Pharmacology, Faculty of Medicine & Allied Sciences, Rajarata University of Sri Lanka, Saliyapura 50008, Sri Lanka; d Department of Medicine, Faculty of Medicine & Allied Sciences, Rajarata University of Sri Lanka, Saliyapura 50008, Sri Lanka; e Clinical Pharmacology Unit, University of Edinburgh, The Queen's Medical Research Institute, 47 Little France Crescent, Edinburgh EH16 4TJ, UK; f Division of Morphology, Centre of Toxicology Sciences and Research, Medical School, University of Crete, Crete, Greece

**Keywords:** Pesticides, Dialkylphosphates metabolites, Hair, Sri Lanka, Chronic exposure

## Abstract

Measuring chronic pesticide exposure is important in order to investigate the associated health effects. Traditional biological samples (blood/urine) are difficult to collect, store and transport in large epidemiological studies in settings such as rural Asia. We assessed the acceptability of collecting hair samples from a rural Sri Lankan population and found that this method of data collection was feasible. We also assessed the level of non-specific metabolites (DAPS) of organophosphate pesticides in the hair samples. The median concentration (pg/mg) of each DAP was: diethyl phosphate: 83.3 (IQI 56.0, 209.4); diethyl thiophosphate: 34.7 (IQI 13.8, 147.9); diethyl dithiophosphate: 34.5 (IQI 23.4, 55.2); and dimethyl phosphate: 3 (IQI 3, 109.7). Total diethylphosphates were recovered in >80% of samples and were positively correlated with self-reported pesticide exposure.

## Highlights

• Collecting hair samples from a rural Sri Lankan population is feasible.
• Organophosphate metabolites were positively correlated with pesticide exposure
• Diethylphosphate levels were higher in Sri Lanka compared to other published studies.

## 1 Introduction

It is estimated that nearly 2.8 million tons of pesticides are used each year globally Ecobichon (2001). Farmers in developing countries increasingly rely on these chemicals Ecobichon (2001). In low and middle income countries, like Sri Lanka, the majority of farmers use pesticides as the main pest management practice; applying pesticides at a concentration 35% higher than recommended, often without following safe practices Nagenthirarajah and Thiruchelvam (2010). In addition the improper disposal, storage and cleaning of spraying equipment is likely to increase environmental contamination and consequently increase pesticide exposure of non-farming individuals. Therefore the level of chronic (long-term) exposure of pesticides may be higher in these settings than in high income countries.

It is not clear whether individuals in developing countries experiencing this chronic exposure are at increased risk of ill health. Acute exposure to pesticides has been associated with negative health effects, such as birth defects, cancer, respiratory and neurological disease, infertility and death Jeyaratnam, 1990, Michalakis et al., 2014, Kanavouras et al., 2011, Baltazar et al., 2014, Mehrpour et al., 2014, Zaganas et al., 2013.Investigations into the health effects of chronic exposure are limited because traditional biological assays like blood and urine only indicate short-term exposure and are difficult to collect, store and transport in large population studies Kavvalakis and Tsatsakis, 2012, Koutroulakis et al., 2014, especially in developing countries. An alternative is to use hair samples, these provide a measure of longer term exposure (dependent on hair length). Studies in European populations have shown that it is possible to assess pesticide exposure using non-specific dialkyl phosphate metabolites (DAPs) using hair samples from the general population Tsatsakis et al., 2010, Kokkinaki et al., 2014. In Sri Lanka, hair is believed to be used in sorcery practices (*gurukam*), and hair sampling is not a routine procedure, therefore collection in this setting maybe problematic Senarthna (2014). The aim of this study was to assess the feasibility and acceptability of obtaining hair samples from people living in rural Sri Lanka, and to use these samples to assess long-term exposure to pesticides by measuring levels of non-specific metabolites (DAPs) of organophosphate pesticides to inform future epidemiological studies.

## 2 Methods

### 2.1 Sampling

This cross-sectional feasibility study was based in the North Central Province of Sri Lanka in a rural village with a mixture of agricultural and non-agricultural households. This area has both rice and vegetable farmers, and therefore represents a village with moderate/high levels of chronic exposure. Using key informants we invited villagers to a public meeting to describe the study. Participants were recruited via home visits and we purposively sampled 50 adults (≥18 years) in order to collect data from broad age and gender categories during the area′s dry season (Yala).The amount of pesticide used in the Yala season by rice farmers (and hence acute exposure) is likely to be less than during the main rice cultivation season (Maha) Konradsen et al. (2007).

Participants were asked to give a hair sample of a pencil width diameter from the back of their head. For individuals with limited head hair we collected chest, arm or leg hair. We also collected demographic details and self-reported information about pesticide exposure.

To assess the feasibility and acceptability of hair sampling, we asked participants for feedback on their experience. Participants were given a 750 Rs (5 USDollars) shopping voucher for their participation. To ensure that the voucher did not impact on the informed consent process, participants were given an information sheet to read, but the gift voucher was not highlighted by researchers until the end of the interview. The samples were analysed by the laboratory of Toxicology and Forensic Chemistry, University of Crete Medical School, Greece.

Organophosphates (OPs) are commonly used in this area and their mode of action is by targeting the nervous system of pests, a system that is also shared by humans. Samples were analysed for DAP metabolites of OPs: dimethyl phosphate (DMP), diethyl phosphate (DEP), diethyl thiophosphate (DETP), and diethyl dithiophosphate (DEDTP). These metabolites are frequently used as biomarkers for OPs exposure in humans and reflect long-term exposure to Ops Kavvalakis and Tsatsakis, 2012, Margariti and Tsatsakis, 2009a, Maravgakis et al., 2012, Margariti and Tsatsakis, 2009b, Tsatsakis et al., 2009. The parent pesticide compounds of these DAP metabolites are summarised in (Table 1).

### 2.2 Laboratory analysis

#### 2.2.1 Materials

The DAPs were purchased from Acros Organics (Geel, Belgium, New Jersey, USA) (dimethylphosphate 98%), from Chem Service (West Chester, USA) (diethylphosphate 98.9%) and from Sigma-Aldrich (USA) (diethylthiophosphate 98%, diethyldithiophosphate 95% and acetonitrile-LCMS grade). Toluene and potassium carbonate (K_2_CO_3_) were obtained from Merck (Darmstadt, Germany). Dibutyl phosphate (DBP used as internal standard) was obtained from Roth (Karlsruhe, Germany).

#### 2.2.2 Standard and spiked solutions

The standard solutions of mixed DAPs were prepared in methanol (0–1000 ng/ml) and kept at 4 °C. Human hair samples with levels of DAPs lower than the limit of quantification (LOQ) values were pooled and used for the preparation of the spiked samples (0, 50, 100, 250, 500 and 1000 pg/mg).

#### 2.2.3 Extraction of dialkylphosphate metabolites

The analytical procedure for the extraction of DAPs from the hair samples has been published previously Tsatsakis et al., 2010, Tsatsakis et al., 2012. Briefly, the decontamination step was done by washing hair samples twice with water and methanol. The dried hair samples were cut (in mm) and an amount of 100 mg was transferred in a test-tube where 2 ml of methanol and 100 ng of DBP (IS) were added. The extraction of the metabolites was performed by incubation of the samples in an ultrasonic bath for 4 h at 50±5 °C. After that, liquid-solid extraction was performed for 30 min. The mixture was centrifuged at 4000 rpm for 5 min and the supernatant was filtered, 15 mg of K_2_CO_3_ and 50 mg Na_2_S_2_O_5_ were added and evaporated to dryness. In the dry residue, 1 ml of acetonitrile, 15 mg of K_2_CO_3_ and 0.1 ml solution of pentafluorobenzylbromide (PFBBr) in acetonitrile (1:3 v/v) were added and incubated at 80 °C for 30 min in a water bath. When the derivatization procedure was completed and the evaporation was done the residue was re-dissolved in 50 μl of toluene.

#### 2.2.4 Gas chromatography and Mass spectrometry conditions

Analysis of the samples was performed by a GC MS QP-2010 Shimadzu system, while the separation of the analytes was done by a Supelco Analytical SLBtm-5 ms (Bellefonte PA, USA) column of 30 m length, 0.25 mm i.d, 0.25 µm film thickness. The flow rate of helium was 1 ml/min while 2 μl of the solution was injected in the splitless mode. The temperature program started from 70 °C for 1 min, raised with a rate of 5 °C/min to 210 °C and then to 350 °C with a rate of 35 °C/min. The injector, interface and ion source temperatures were set at 270 °C, 300 °C and 230 °C, respectively. The retention time of each metabolite was 15.7, 17.9, 21.4, 23.0 and 25.04 min for DMP, DEP, DETP, DEDTP and DBP, respectively. The determination and quantification of the analytes was achieved in selected ion monitoring (SIM) using m/z 110, **306** for DMP, **258**, 334 for DEP, **350**, 274 for DETP, **366**, 185 for DEDTP and **335** for DBP (in bold the ions were used for the quantification).

### 2.3 Statistical analysis

Concentrations of DMP, DEP, DETP and DEDTP were summarised using medians and interquartile interval (IQI) by factors relating to demographic, sample, and self-reported pesticide exposure characteristics. All values that were below the level of determination (LOD) were set to LOD/2.The LOD for each DAP was estimated as follows: 6 pg/mg for DMP; 5 pg/mg for DEP and DETP; and 3 pg/mg for DEDTP. The total level of DEP, DETP, DEDTP and DMP was compared for each self-reported pesticide exposure. Due to the high percentage of readings lower than the LOD for DMP we also compared the total level of diethylphosphates (DEP, DETP, and DEDTP) in order to inform our conclusion. As this was a feasibility study we did not conduct any formal statistical tests on the data.

## 3 Results

### 3.1 Sampling

The response rate for this study was 96% (50/52); a male in his 50 s and an elderly female refused to take part in the study. As part of the study we asked participants for feedback about their experience. Participants indicated that they thought that understanding pesticide exposure and the associated health effects in their population group was very important. They felt the use of key informants and the public meeting were imperative for the success of a future project in order to reduce doubt about what the samples were to be used for. One participant indicated that for future work a joint male and female data collection team would be needed to collect samples from female members particularly when male household members were absent. They felt an unknown male handling a female′s hair may lead to later domestic problems. An important findings for future work was that the majority of participants indicated that the shopping voucher undermined their voluntary contribution. They indicated that it would have been more appropriate to have been given a memento (e.g. a statue) of their participation that they could display in their homes.

### 3.2 Monitoring of DAPs in hair samples

The levels of DAPs found in the hair samples of individuals living in rural Sri Lanka are summarised in Table 2, Table 3. A large proportion (58%) of samples were below the LOD for DMP. The median concentration (pg/mg) of each DAP in hair samples was: DEP: 83.3 (IQI 56.0, 209.4); DETP: 34.7 (IQI 13.8,147.9); DEDTP: 34.5 (IQI 23.4, 55.2); and DMP:3 (IQI 3,109,7). The median level of total diethylphosphates was higher in individuals who reported higher levels of pesticide exposure (e.g. farmers and sprayers had higher levels compared to non-farmers/sprayers) (Table 3).

## 4 Discussion

The findings of this study indicate that it is feasible to collect hair samples for biomarker research purposes in rural population in Asia, despite the cultural significance of hair in this setting. DEP, DETP and DEDTP had detection rates of over 80%, but DMP was only detectable in 42% of samples. We observed differences in median concentration of total diethylphosphates with self-reported measures of pesticide exposure e.g. occupation and pesticide spraying activities.

Compared to another study with a similar population of individuals involved in spraying in Greece (n=34), the median levels of diethylphosphates in sprayers were 9 and 2 times higher for DEP and DETP (respectively) in the current study. The non-sprayers in the Sri Lankan sample had 5–8 times higher median diethylphosphate levels (DEP & DETP) than non-sprayers in Greece. A previous study in Crete also found variation in the DAP values detected, with lower levels and frequencies of detection of DMP compared to the other DAP metabolites in occupational exposed population [11]. The authors conclude that the lower level or frequencies of detection was due to the parent organophosphate compounds not being used in high concentrations in the study area. This could also explain the low detection rate of DMP in rural Sri Lanka (Table 1). It could also be due to lower levels of expression or activity of enzymes which metabolise the parent organophosphates of DMP in this Asian population.

To the best of our knowledge this is the first study assessing the feasibility of collecting general population hair samples for research purposes in Sri Lanka. The findings of this study indicate that it is possible to collect hair samples for biomonitoring purposes in rural Sri Lanka. Whilst not all DAPs were detected to a high level in this sample, the recovery of diethylphosphates indicated a correlation between levels of these metabolites and pesticide exposure. We only analysed total exposure to pesticides and did not perform a segmental analysis of hair. A segmental analysis would have allowed us (especially for longer hair samples) to measure an individual′s exposure during different periods of time. The findings of this study will be important to inform the conduct of future epidemiological studies in this region.

## Funding

This work was supported by the Wellcome Trust [WT099874MA]. DG is a National Institute for Health Research Senior Investigator.

## Ethical statement

Ethical approval was obtained from the Ethical Review Committee of the Faculty of Medicine and Allied Sciences, Rajarata University of Sri Lanka (ERC/2014/046).

## Figures and Tables

**Table 1:** Studied dialkyl phosphate metabolites and the parent organophosphate pesticides.

Parent pesticide compounds	Dialkyl phosphate metabolites			
DMP	DEP	DETP	DEDTP
Azinphos-Methyl	x			
Chlorethoxyphos		x	x	
Chlorpyrifos		x	x	
Chlorpyrifos-Methyl	x			
Coumaphos		x	x	
Diazinon		x	x	
Dichlorvos	x			
Dicrotophos	x			
Dimethoate	x			
Disulfoton		x	x	x
Ethion		x	x	x
Ethylparathion			x	
Fenitrothion	x			
Fenthion	x			
Isazofos-Methyl	x			
Malathion	x			
Methidathion	x			
Methylparathion	x			
Mevinphos	x			
Naled	x			
Oxydemeton-Methyl	x			
Parathion		x	x	
Phorate		x	x	x
Phosalone		x	x	x
Phosmet	x			
Primiphos-Methyl	x			
Sulfotepp		x	x	
Temephos	x			
Terbufos		x	x	x
Tetrachlorvinphos	x			
Tribufos		x	x	
Trichlorfon	x			

Dimethyl phosphate (DMP), diethyl phosphate (DEP), diethyl thiophosphate (DETP), and diethyl dithiophosphate (DEDTP).

**Table 2:** Median and interquartile interval of metabolite levels (pg/mg) in hair samples by demographic and sample characteristics.

	Median (interquartile interval) pg/mg				
	n	DEP	DETP	DEDTP	DMP
**Sample characteristics**					
% Positive samples^a^	50	82%	90%	82%	42%
Median (IQI)	50	83.3 (56.0,209.4)	34.7 (13.8,147.9)	34.5 (23.4, 55.2)	3.0 (3.0,109,7)
Type of hair					
Chest/arm/leg	4	166.0 (97.5,678.9)	164.6 (57.6,312.4)	61.0 (40.0,131.8)	3 (3.0,3.0)
Head	46	80.7 (56.0,209.4)	31.4 (13.1,82.5)	34.1 (22.1,53.3)	3.0 (3.0,146.2)
Hair length (cm)					
0–1	11	76.7 (2.5,184.9)	40.0 (2.5,82.5)	52.1 (29.9,193.5)	3.0 (3.0,84.8)
2–7	10	204.7 (66.6,311.4)	95.2 (16.8,201.6)	34.2 (23.6,85.0)	3.0 (3.0,521.0)
8–12	14	81.0 (60.5,620.9)	27.9 (11.6,185.3)	31.4 (1.5,55.2)	7.5 (3.0,412.4)
13–22	13	76.9 (53.8,93.9)	33.4 (22.6,66.1)	32.6 (25.7,35.6)	3.0 (3,146.2)
**Demographic details**					
Sex					
Female	27	73.9 (53.8,301.1)	22.6 (10.8,181.6)	35.6 (23.6,85.0)	3.0 (3.0,75.4)
Male	23	128.4 (62.1,209.4)	47.7 (27.0,147.9)	32.6 (1.5,52.9)	11.9 (3.0,243.2)
Age group					
18–25	7	76.9 (2.5,184.9)	71.6 (2.5,95.7)	32.6 (1.5,52.1)	28.0 (3.0,854.4)
26–35	12	78.8 (59.8,171.1)	30.8 (17.3,104.9)	27.9 (11.8,36.1)	3.0 (3.0,3.0)
36–55	18	94.7 (62.1,171.0)	43.8 (19.0,147.9)	36.3 (27.9,70.0)	43.5 (3.0,146.2)
56–65	5	66.6 (60.5,76.7)	11.7 (6.1,29.4)	30.1 (26.6,41.1)	25.8 (3.0,109.7)
66+	8	347.0 (100.4,833.2)	142.1 (14.3,348.8)	123.2 (35.8,231.8)	3.0 (3.0,16.9)
SEP (quality of household building)^b^					
Low	6	140.8 (2.5,301.1)	51.0 (2.5,181.6)	93.3 (29.9,205.1)	3.0 (3.0,75.2)
Middle	28	87.7 (63.7,296.2)	48.3 (11.6,154.4)	31.9 (11.3,43.4)	3.0 (3.0,194.7)
High	15	78.5 (48.0,147.1)	29.4 (19.5,95.7)	38.2 (27.9,70.0)	3.0 (3.0,208.2)

a ≪LOD were set to LOD/2.

b Construction materials used for roof, walls and floor were used to categorise households into these groups. We used a similar method to that used by the Sri Lankan census.

**Table 3:** Median and interquartile interval of metabolite levels (pg/mg) by self-reported pesticide exposure.

	Median (interquartile interval) pg/mg						
	n	DEP	DETP	DEDTP	DMP	Total DEPs*	Total DAP**
Occupation							
Other	14	80.2 (2.5,147)	24.8 (13.1,71.6)	34.5 (22.1,70.0)	3.0 (3.0,243.2)	171.9 (91.1,332.7)	346.6 (94.1,582.3)
Farming	18	133.7 (62.1,311.4)	46.7 (19.5,161.0)	30.6 (21.5,51.6)	19.9 (3.0,146.2)	208.9 (144.0,532.9)	385.7 (194.8,780.0)
Housewife	18	70.3 (60.5,301.1)	34.7 (10.8,181.6)	36.9 (25.7,63.3)	3.0 (3.0,30.8)	140.0 (99.3,567.7)	224.6 (123.5,996.6)
Involved in pesticide spraying (wet/maha season)							
1–3 Times a week	6	197.2 (132.7,311.4)	121.8 (29.4,161.0)	30.6 (1.5,45.7)	3.0 (3.0,3.0)	364.5 (191.8,473.9)	438.0 (335.7,708.9)
2–3 Times a month	14	71.0 (48.0,281.0)	28.4 (19.1,82.5)	32.0 (23.4,52.9)	7.5 (3.0,106.7)	125.3 (95.2,532.9)	276.2 (100.8,780.0)
Once a month	5	65.4 (2.5,171.0)	36.0 (16.8,53.5)	38.4 (1.5,193.5)	3.0 (3.0,3.0)	226.0 (120.5,258.0)	261.0 (123.5,372.2)
Never	25	76.9 (60.5,147.0)	33.4 (11.7,76.3)	35.6 (25.7,63.3)	3.0 (3.0,208.2)	151.8 (109.6,354.6)	346.2 (132.5,879.8)
Involved in pesticide spraying (dry/Yala season)							
1–3 Times a week	6	133.7 (128.4,209.4)	45.7 (27.9,161)	15.7 (1.5,30.1)	54.8 (3.0,521.0)	209.2 (187.9,396.2)	438.0 (333.2,708.9)
2–3 Times a month	15	63.6 (2.5,281.0)	28.7 (11.6,95.7)	37.4 (23.4,52.1)	3.0 (3.0,75.2)	137.9 (95.1,532.9)	219.2 (98.4,570.7)
Once a month	4	118.2 (33.9,671.9)	44.8 (26.4,222.4)	116.0 (20.0,206.5)	3.0 (3.0,74.6)	242.0 (173.3,1007.8)	316.6 (192.2,1066.4)
Never	25	83.0 (60.5,147.1)	36.0 (11.7,233.6)	35.6 (25.7,70.0)	3.0 (3.0,109.7)	170.0 (109.6,450.6)	357.6 (132.5,996.6)
Use of non-agricultural pesticides							
No	8	100.7 (64.4,207.8)	38.5 (12.3,131.8)	30.0 (24.3,53.1)	55.3 (7.5,313.9)	180.9 (97.2,381)	295.9 (116.1,1151.8)
Yes	42	83.3 (48.0,209.4)	34.7 (16.8,147.9)	35.2 (23.4,63.3)	3.0 (3.0,109.7)	164.4 (106.6,450.6)	351.9 (132.5,780.0)
Distance to nearest field (m)							
≤5	12	133.7 (84.6,260.4)	42.4 (23.5,173.1)	30.6 (28.9,212.3)	3.0 (3.0,54.8)	242.3 (179.6,435.0)	345.4 (184.6,592.9)
6–200	13	78.5 (53.8,184.9)	21.5 (6.1,95.7)	27.9 (21.0,51.6)	25.8 (3.0,208.2)	137.9 (95.4,332.7)	346.2 (125.1,1114.5)
201–365	12	139.6 (68.8,318.0)	46.7 (18.2,233.9)	35.9 (24.6,93.3)	3.0 (3.0,110.8)	198.0 (126.8,595.6)	471.4 (165.9,1110.6)
366–500	13	62.1 (2.5,76.9)	36.0 (13.1,71.6)	35.6 (22.1,53.3)	3 (3.0,109.7)	142.2 (91.1,181.1)	258.7 (107.1,453.6)

* Total diethylphosphates (DEP+DETP+DEDTP).

** Total non-specific dialkyl phosphate (DEP+DETP+DEDTP+DMP).
